# Developing and Validating a Method for Separating Flavonoid Isomers in Common Buckwheat Sprouts Using HPLC-PDA

**DOI:** 10.3390/foods8110549

**Published:** 2019-11-04

**Authors:** Davin Jang, Young Sung Jung, Mi-Seon Kim, Seung Eel Oh, Tae Gyu Nam, Dae-Ok Kim

**Affiliations:** 1 Graduate School of Biotechnology, Kyung Hee University, Yongin 17104, Korea; davin1031@khu.ac.kr (D.J.); miseonkim95@khu.ac.kr (M.-S.K.); 2 Department of Food Science and Biotechnology, Kyung Hee University, Yongin 17104, Korea; chembio@khu.ac.kr; 3 Korea Food Research Institute, Wanju 55365, Korea; dr51@kfri.re.kr

**Keywords:** common buckwheat sprout, flavonoid isomer, quercetin-3-*O*-robinobioside, validation, chromatographic separation

## Abstract

Buckwheat sprouts that are synthesized during the germination process are rich in flavonoids, including orientin, vitexin, rutin, and their isomers (isoorientin, isovitexin, and quercetin-3-*O*-robinobioside, respectively). The purpose of this study was to optimize and validate an analytical method for separating flavonoid isomers in common buckwheat sprout extract (CSE). Factors, such as range, linearity, precision, accuracy, limit of detection, and limit of quantification, were evaluated for each standard using high-performance liquid chromatography (HPLC). On the basis of resolution and symmetry, a column temperature of 40 °C with 0.1% (*v*/*v*) acidic water and acetonitrile as mobile phases, at a flow rate of 1 mL min^−1^ were determined to be the optimal analytical conditions. Calibration curves for orientin, isoorientin, vitexin, isovitexin, and rutin exhibited good linearity with correlation coefficients of 0.9999 over the 6.25–100.00 μg mL^−1^ range. Recovery values of 96.67–103.60% confirmed that the method was accurate for all flavonoids. The relative standard deviations of intra-day repeatability and inter-day reproducibility confirmed method preciseness, with values of less than 5.21% and 5.40%, respectively. The developed method was used to analyze flavonoids in CSE, with isomers satisfactorily separated and simultaneously quantified. We demonstrated that the developed HPLC method can be used to monitor flavonoids in buckwheat sprouts.

## 1. Introduction

Buckwheat is a pseudocereal belonging to the *Polygonaceae* family that grows rapidly and is tolerant to cold [1]. Buckwheat is found almost everywhere but is mainly grown in the northern hemisphere [2]. Common buckwheat (*Fagopyrum esculentum* Möench) and tartary buckwheat (*F. tataricum* Gaertner) are the most consumed buckwheat species [1]. Among the edible parts of common buckwheat, the sprouts have attracted considerable attention in recent years [3] as they are considered to be a popular health food and are widely consumed because of their bioactive compounds [4,5]. The sprout-germination process induces the hydrolysis of triglycerides in the seeds and produces the energy required for various biochemical reactions through the tricarboxylic acid cycle [6], and the content of bioactive compounds in the seeds increases through chemical reactions, for example, the flavone glycoside content is known to increase during the germination of common buckwheat [7,8]. Common buckwheat sprout (CS) has been studied for a variety of pro-health benefits, such as its antioxidant capacity [7,9] and anti-inflammatory effects [10,11].

CS has been reported to have more abundant flavone *C*-glucosides than those of the tartary species [12]. The flavone *C*-glucosides in CS are present as orientin and isoorientin, from luteolin as the parent, as well as vitexin and isovitexin, from apigenin as the parent (Figure S1). In addition, rutin is a representative flavonoid present in common buckwheat and CS [12]. A recent report revealed that quercetin-3-*O*-robinobioside (Q3R), which is an isomer of rutin, exists in CS [8]. Q3R is known to be present in cotyledon and immature common buckwheat, rooibos, mature saskatoon fruit, and jujube fruit [8,13,14,15]. To summarize, the six main flavonoids found in CS exist as three sets of isomer pairs, with differences due to the position and form of the sugar.

Flavonoids and their glycosides can be used as quality control markers for many phytomedicines and medicinal plants [16]. Various analytical techniques have been developed for the separation and detection of flavonoid glycosides, in which the most-widely employed method is reversed-phase high-performance liquid chromatography (HPLC), coupled with photodiode array (PDA) detection and/or mass spectrometry (MS) [14,16,17]. Qualitative analysis of flavonoids is possible using MS even when baseline separation has not been secured. However, structural isomers cannot be distinguished only on the basis of MS/MS information because they have the same molecular weight and similar fragment patterns [16,18,19]. Therefore, flavonoid glycosides need to be separated in order to enable their accurate quantitative analysis in food. Previous studies reported the effects of column temperature, composition of mobile phase, and flow rate on the separation of a flavonoid isomer for qualitative analysis [20,21]. Even with a column temperature of 40 °C and the addition of acid to solvents, orientin and Q3R in CS were not separatable with isoorientin and rutin, respectively [7,9]. Q3R and rutin in mature saskatoon and jujube fruits were not completely separated and were not suitable for quantification [13,22]. Therefore, a suitable HPLC method for quantitatively analyzing flavonoid isomers present in CS is necessary.

In this study, we introduce an HPLC analysis method that simultaneously quantifies two types of flavone-*C*-glycoside isomer and flavonol-*O*-glycoside isomer found in common buckwheat sprout extract (CSE). The HPLC conditions of the developed analytical method, including mobile phase, column temperature, and flow rate, were optimized. The developed method was validated by determining the range, linearity, precision, accuracy, limit of detection (LOD), and limit of quantification (LOQ) for each compound.

## 2. Materials and Methods

### 2.1. Chemicals and Reagents

Orientin, isoorientin, vitexin, and isovitexin (all with ≥99% purity) were purchased from Extrasynthese (Genay, France). Rutin hydrate (≥94%), HPLC-grade formic acid (≥98%), and dimethyl sulfoxide (DMSO) were obtained from Sigma Aldrich Co., LLC (St. Louis, MO, USA). HPLC-grade water, methanol, and acetonitrile were purchased from Thermo Fisher Scientific (Waltham, MA, USA).

### 2.2. CSE Preparation

CS seeds were purchased from First Village Farmer’s Union under Hallasan Mountain (Jeju-do, Republic of Korea) in 2018. Seeds were planted for 6 days at 25 °C in a dark growth chamber. Sprouts were harvested and freeze-dried (FD 8518; Ilshin Lab Co., Ltd., Dongducheon, Korea) for 3 days. The CSE was obtained by mixing the dried buckwheat sprouts with 90% (*v*/*v*) aqueous methanol. The mixture was ultrasonicated for 30 min and the supernatant was acquired by centrifugation (2232× *g*) for 10 min and filtered through a 0.45-μm polyvinylidene fluoride syringe filter (Millipore, Billerica, MA, USA). The residue was re-extracted once using the procedure described above. The filtrate was evaporated on a rotary evaporator (N-1000; Eyela, Tokyo, Japan) in a water bath at 40 °C. The extract was stored in a deep freezer (WSM-2700UC; Grand Woosung Inc., Seoul, Korea) at −50 °C and freeze-dried (FD 8518) for 3 days. All experiments were performed in triplicate.

### 2.3. Flavonoids Analysis by Reversed-Phase HPLC

The 3 sets of isomer pairs were analyzed by modifying an existing method [8]. CSE and its flavonoids were analyzed using HPLC (Alliance e2695; Waters, Milford, MA, USA) with the Empower 3 software (Waters), a PDA detector (2998, Waters), and a ProntoSIL 120-5-C18-ace-EPS column (4.6 × 250 mm, 5.0 μm; Bischoff, Leonberg, Germany), with the flavonoids and extract monitored at 360 nm. The column temperature was set to 40 °C and an injector volume of 5 μL was used. Gradient elution was carried out with 0.1% (*v*/*v*) formic acid in water (solvent A) and acetonitrile (solvent B). All solvents were filtered and degassed. A flow rate of 1.0 mL min^−1^ was used. The following binary mobile-phase linear gradients were used: 100% A at 0 min, 90% A at 4 min, 86% A at 20 min, 84% A at 30 min, 84% A at 36 min, 80% A at 44 min, 80% A at 50 min, 75% A at 54 min, 30% A at 58 min, 30% A at 62 min, 15% A at 66 min, 15% A at 70 min, 100% A at 72 min, and 100% A at 75 min. Flavonoids were identified by comparing their retention time and ultraviolet (UV) spectra with those of their respective standards. The Q3R was identified as in previous studies and was quantified using the calibration curve for rutin because the standard for Q3R is not commercially available [13,22].

### 2.4. Optimizing the Chromatography Conditions

The conditions for the separation of the flavonoids in CSE include the mobile phase, temperature, and flow rate. Water or 0.1% (*v*/*v*) formic acid in water as solvent A and methanol or acetonitrile as solvent B were used. The column temperatures were set to 20, 30, and 40 °C. The separation of the 3 sets of isomer pairs was evaluated. Flow rates of 0.6, 0.8, and 1.0 mL min^−1^ were used. The column and method used are listed in Section 2.3. The resolution (*Rs*) and symmetry factor were calculated as follows [23]:

*Rs* = 1.18 × (t_R2_ − t_R1_)/(W_2_ + W_1_)

where t_R2_ − t_R1_ is the difference in retention time and W_2_ + W_1_ is the sum of the peak widths at half of peak height, and,

*Symmetry factor* = W_0.05h_/2*f*

where W_0.05h_ is the peak width at 1/20th the peak height above the peak baseline, and *f* is the distance along the horizontal line drawn from the leading edge of the peak to the vertical line drawn from where the peak dissects a horizontal line drawn at 1/20th of the peak height above the peak baseline (W_0.05h_).

### 2.5. Method Validation

The validation criteria are based on the guidelines published by the Ministry of Food and Drug Safety [23]. Spectral scans of peaks were performed with the PDA detector (2998, Waters) in order to verify method specificity. The linearity of each calibration curve, range, precision, accuracy, LOD, and LOQ were also evaluated.

#### 2.5.1. Linearity

Stock solutions of standard compounds in CSE were prepared at a concentration of 10 mg mL^−1^ in DMSO and 10% (*v*/*v*) DMSO in methanol was used to dilute the stock solution to the appropriate concentration. The concentration ranges of the standard compounds were appropriately set to include the CSE to be analyzed through preliminary experiments. Linearity ranges were determined by diluting the standard solutions to six different concentrations. The method was found to be linear in 6.25–100.00 μg mL^−1^ range for all standard compounds. Each concentration was analyzed in triplicate. The analytical curve was obtained from the peak area corresponding to each standard compound at six different concentrations. The linearity of each calibration curve is expressed by its correlation coefficient (R).

#### 2.5.2. Accuracy, Precision, and Recovery

Accuracy was evaluated as the percent recovery (%) at three concentrations (25.00, 50.00, and 100.00 μg mL^−1^) of spiked standard solutions and blanks (dilution solvent). Precision is expressed as the relative standard deviation (RSD) of intra-day repeatability (analysis was performed on the same day (*n* = 3) with the same instrument) and inter-day reproducibility (three different days (*n* = 3 × 3) using the same instrument).

#### 2.5.3. Limits of Detection and Quantification

LODs and LOQs for the analyte flavonoids were calculated from the standard deviation of the response and the slope of the calibration curve. The standard curves were constructed in the 0.61–20.00 μg mL^−1^. The LOD was calculated as 3.3 × σ/s (σ = standard deviation of the response, s = slope of the standard curve), while 10 × σ/s was used for the LOQ.

### 2.6. Statistical Analysis

Analysis was performed in triplicate and the results obtained are represented as means ± standard deviations. One-way analysis of variance followed by the Tukey’s test (*p* < 0.05) was applied to determine the significances of the differences among the means. Tests for statistical significance were performed using IBM SPSS software (Version 23; IBM SPSS Statistics Inc., Armonk, NY, USA).

## 3. Results and Discussion

### 3.1. Effects of Elution Conditions on Isomer Separation

The mobile phase, temperature, and flow rate are significant isomer-separation factors in HPLC [24,25]. The data for the various isomers in CSE using different mobile phases are listed in Table 1, at a column temperature of 40 °C and a flow rate of 1.0 mL min^−1^. *Rs*, a quantitative value that indicates the degree of separation between adjacent components should be greater than 1.5 in order to satisfy the baseline-separation criteria [23]. The data listed in Table 1 are for water as solvent A and an organic solvent as B. Luteolin, apigenin, and quercetin derivatives include orientin and isoorientin, vitexin and isovitexin, and Q3R and rutin, respectively, and the *Rs* values for the luteolin and quercetin derivatives were less than 1.5 when methanol was used as solvent B. We conclude that methanol is not suitable as solvent B. On the other hand, all components were completely separated using acetonitrile, which has a higher elution strength than methanol and is universally used due to its UV cutoff and viscosity [25]. Symmetry factors were calculated in order to determine whether or not proton (hydrogen ion) donor needs to be added to the water that accompanies the acetonitrile. Peaks appear to be more symmetric as the symmetry factor approaches unity [23] and values in the 0.99–1.03 range were observed for all components using acidic water as mobile phases (Table 1). Peaks were not completely separated when methanol was used as a mobile phase, which means that symmetry factors could not be calculated. Therefore, the optimal mobile phases for analyzing CSE are water containing 0.1% (*v*/*v*) formic acid as solvent A and acetonitrile as solvent B.

**Figure 1:**
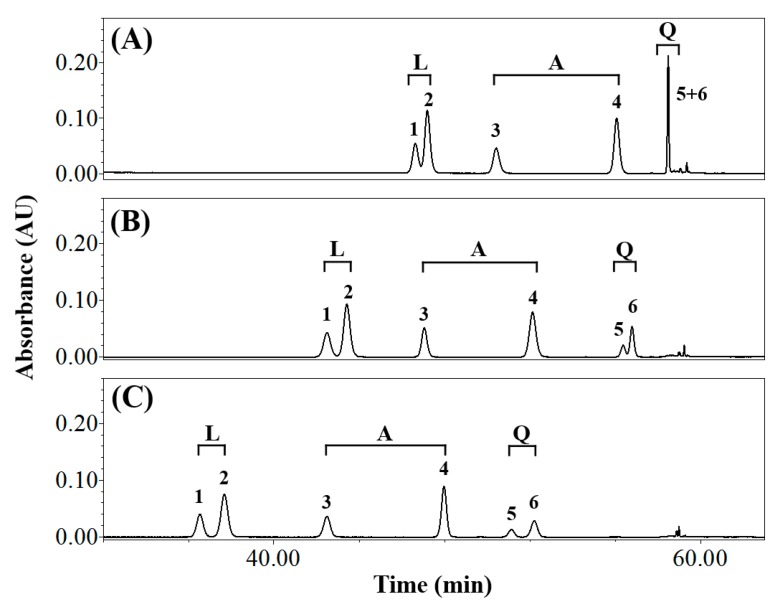
Reversed-phase high-performance liquid chromatography (HPLC) traces of common buckwheat sprout extract acquired at: (**A**) 20 °C, (**B**) 30 °C, and (**C**) 40 °C detected at 360 nm. Peak number: 1, orientin; 2, isoorientin; 3, vitexin; 4, isovitexin; 5, quercetin-3-*O*-robinobioside; 6, rutin. L, luteolin derivatives; A, apigenin derivatives; Q, quercetin derivatives.

The chromatogram shown in Figure 1 reveals the effect of temperature on isomer separation. The calculated *Rs* values are listed in Table S1. The above-mentioned optimal mobile phases were used at a flow rate was 1.0 mL min^−1^. The *Rs* values for the isomer sets were 1.05 (luteolin derivatives), 10.83 (apigenin derivatives), and 0.00 (quercetin derivatives) at 20 °C (Figure 1A and Table S1). Only vitexin and isovitexin (the apigenin derivatives) satisfied the baseline-separation criterion at 20 °C. The luteolin derivatives and apigenin derivatives exhibited *Rs* values of 1.58 and 9.64 at 30 °C, which satisfy the baseline-separation criterion (Figure 1B and Table S1). The *Rs* value of Q3R and rutin (the quercetin derivatives) at 30 °C was 1.15, which clearly does not satisfy the baseline-separation criterion. In contrast, Figure 1C and Table S1 reveal that all isomer sets were baseline separated at 40 °C, with *Rs* values of 1.87, 10.30, and 1.93 for the luteolin, apigenin, and quercetin derivatives, respectively. The analytes also eluted faster as the column temperature was increased from 20 °C to 40 °C as higher temperatures result in lower mobile-phase viscosities and pressures, which lead to shorter retention times [26]. However, high temperatures do not necessarily guarantee efficient separation [24,27], for example, the *Rs* value of the apigenin derivatives seemed to be independent of the increase in temperature with values of 10.83, 9.64, and 10.30 at 20 °C, 30 °C, and 40 °C, respectively (Figure 1). Therefore, appropriate temperature conditions are very important when developing an analytical method. Based on the above results, all subsequent analyses in this study were carried out at 40 °C.

**Figure 2:**
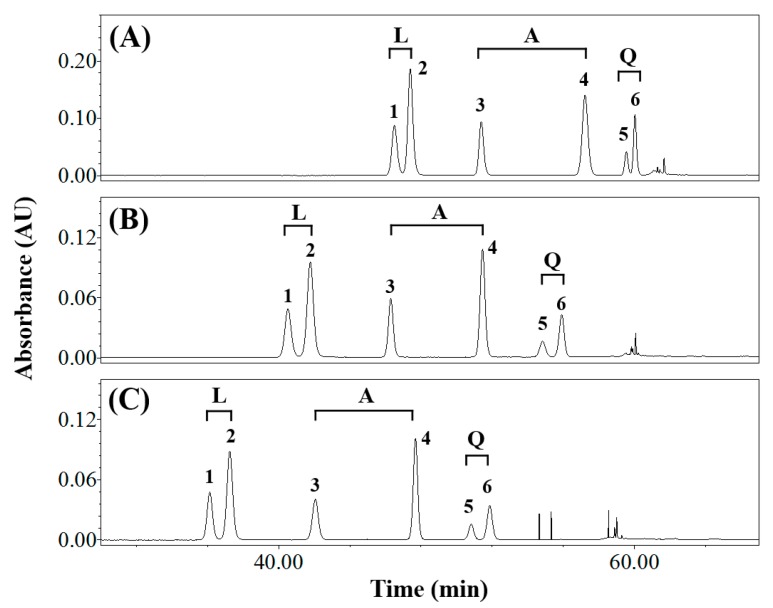
Reversed-phase HPLC traces of common buckwheat sprout extract acquired at flow rates of: (**A**) 0.6 mL min^−1^, (**B**) 0.8 mL min^−1^, and (**C**) 1.0 mL min^−1^ detected at 360 nm. Peak number: 1, orientin; 2, isoorientin; 3, vitexin; 4, isovitexin; 5, quercetin-3-*O*-robinobioside; 6, rutin. L, luteolin derivatives; A, apigenin derivatives; Q, quercetin derivatives.

The effects of flow rate on elution were evaluated at 0.6, 0.8, and 1.0 mL min^−1^ (Figure 2 and Table S2) using the optimal solvents and temperature. All components were separated except for the quercetin derivatives (*Rs* = 1.44) at a flow rate of 0.6 mL min^−1^ (Table S2). The components eluted faster as the flow rate was increased from 0.6 to 1.0 and the flavonoids eluted at 46.52–60.03 min at 0.6 mL min^−1^, but at 36.28–52.00 min at 1.0 mL min^−1^. As a result, a flow rate of 1.0 mL min^−1^ was chosen in subsequent work.

### 3.2. Isolating the Three Isomer Pairs from CS

**Figure 3:**
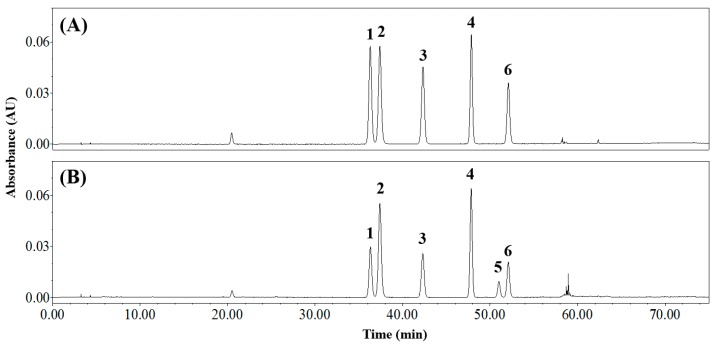
Reversed-phase HPLC traces of (**A**) standards and (**B**) common buckwheat sprout extract. Peak number: 1, orientin; 2, isoorientin; 3, vitexin; 4, isovitexin; 5, quercetin-3-*O*-robinobioside; 6, rutin.

The structures of the three sets of isomer pairs are displayed in Figure S1. Orientin and isoorientin are isomers that have luteolin as their parent, in which a glucose unit is attached at the 8-*C* and 6-*C* position of the flavonoid A ring, respectively. Likewise, vitexin and isovitexin are isomers with apigenin as the parent. Rutin contains a rutinose (6-*O*-α-L-rhamnosyl-D-glucose) at the C-3 position of the quercetin, while Q3R contains a robinobiose (6-*O*-α-L-rhamnosyl-D-galactose) at the same position. Figure 3A shows the ordering of orientin, isoorientin, vitexin, isovitexin, and rutin standards obtained using the optimal HPLC conditions, which is similar to that of a previous CSE analysis result [8]. The -OH of glucose moiety and the -OH of the flavonoid A-ring can interact to form hydrogen bonds [28], resulting in electron distributions and polarities that depend on the position of glucose bonding to the flavonoid (6-*C* or 8-*C*). Therefore, when reversed-phase HPLC is used, the flavone-8-*C*-glycosides elute faster than the corresponding flavone-6-*C*-glycosides [29]. The HPLC trace of the CSE also shows that the flavone-8-*C*-glycosides elute faster than the flavone-6-*C*-glycosides (Figure 3). The Q3R and rutin isomer sets were also detected in the CSE (Figure 3B).

**Figure 4:**
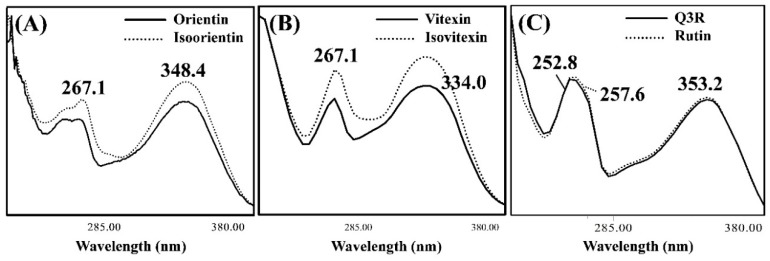
UV spectra of (**A**) orientin and isoorientin, (**B**) vitexin and isovitexin, and (**C**) quercetin-3-*O*-robinobioside (Q3R) and rutin acquired with the HPLC-PDA (photodiode array).

The UV spectra corresponding to the peaks of flavonoids in CSE separated by HPLC-PDA are shown in Figure 4. Band I, which is observed at 300–380 nm, is due to electron transitions in the cinnamoyl group (B-ring of flavonoid backbone), while band II (240–280 nm) corresponds to electron transitions of the benzoyl group (A-ring of flavonoid backbone) [30]. Figure 4A,B show that orientin, isoorientin, vitexin, and isovitexin have the same absorption maximum for bands II (*λ_max_* = 267.1 nm). The sugar attached at the 6-*C* or 8-*C* position of the benzoyl group does not affect the UV spectra. Orientin and isoorientin exhibit band I maxima at 348.4 nm, while those of vitexin and isovitexin are evident at 334.0 nm (Figure 4A,B). Q3R showed band I maxima similar to rutin, while band II maxima was quite different (Figure 4C). The UV spectra of flavonoids are affected by a variety of functional groups (methyl, glycosyl, and hydroxyl) and other molecular features (2,3 double bond and/or a ketone in the C-ring) [31]. For example, -OH groups coupled to the B-ring systematically cause a red-shift of the *λ_max_* of band I [31], with electron delocalization contributing these observations. Consequently, the band I *λ_max_* values of orientin and isoorientin (3´,4´-OH) are higher than those of vitexin and isovitexin (3´-OH). Q3R and rutin exhibit higher band I values (*λ_max_* = 353.2 nm) than the flavones (*λ_max_* = 348.4, 334.0 nm) and the 3-OH present in the flavonol structure reduces π conjugation through stabilization [31].

### 3.3. Validating the Applied Method

#### 3.3.1. Linearity and Range

Calibration curves based on six concentrations were constructed over the 6.25–200.00 μg mL^−1^ range (Table 2). We analyzed five components, however Q3R was not analyzed because no validated standard compound exists. According to guidelines for the validation of analytical procedures for compounds, such as medicines, published by the Ministry of Food and Drug Safety, a response is considered to be linear if the correlation coefficient (R) of the calibration curve exceeds 0.9900 [23]. All calibration curves in this study exhibited good linearities with correlation coefficients over 0.9999, indicating that the responses to the external standards in this study are suitably linear. Therefore, we conclude that a concentration range of 6.25 μg mL^−1^ or higher ensures good linearity when quantifying flavonoids in CSE.

#### 3.3.2. Precision, Accuracy, and Recovery

Validation results, including accuracy and precision, are listed in Table 3. Accuracy and precision were measured for each component using three spiked base-solvent replicates at concentrations of 25, 50, and 100 μg mL^−1^. RSDs of retention times were less than 0.2%. Accuracy is an indication of the degree to which the measured value is close to the true value. The relative area of the main component is set to 100% and the amount of analyte is calculated and expressed as a percent recovery. Values of recovery that ranged between 96.67% and 103.60% were measured for the five components (Table 3). All percent recoveries were within in the 95% confidence interval criterion and were highly accurate. A higher concentration of a spiked standard (25,100 μg mL^−1^) in the blank solvent resulted recovery closer to 100%. We conclude that the method provides values that are close to the true values and is therefore suitable for accurate analysis.

Precision was assessed by the intra-day repeatability and inter-day reproducibility on three different days, and is expressed as a RSD. The RSDs of intra-day repeatability measured three times in a single day were found to be 2.67–5.21% (Table 3). Based on the RSD values of the five flavonoids measured in this study, we showed that the developed analysis method is consistent and precise [32,33].

#### 3.3.3. Limits of Detection and Quantification

The five standard components were analyzed over the 0.61–20.00 μg mL^−1^ range in order to determine LODs and LOQs, which were calculated from the standard deviation of the response and the slope of the calibration curve (Table 3). LOD and LOQ are used to verify the capacity of a method to detect and quantify materials at low concentrations [23]. The results show the concentrations used in the standard curves are appropriate and consistent with previous validation studies [34,35]. These results show that the new method is very sensitive toward the quantification of flavonoids in CSE.

### 3.4. Applying the Analytical Method to CSE

We verified the method using CSE, the results of which are listed in Table 4. The developed HPLC method was used to analyze eight CSEs produced at different batches. All samples were prepared in the same manner (described above). The flavonoids in each extract were detected in the elution order with an increasing retention time as follows: orientin, isoorientin, vitexin, isovitexin, Q3R, and rutin. Except for Q3R, which was detected only in the extract, the t_R_ values of the other five components are consistent with those listed in Table 3. Q3R was detected at 51.1 min, eluting between isovitexin and rutin. All samples exhibited satisfactory validation data, with the calculated LODs and LOQs listed in Table 3. The flavonoid content in the CSEs was calculated based on the standard curves provided in Table 2. There were differences in content per growth lot, but the results show that the content of 6-*C*-glycosyl flavones is higher than that of the flavone-8-*C*-glycosides in the CSEs, as reported previously [11,12]. The results also show that there was more rutin than Q3R in CSEs, in line with reported results [11]. Similar trends were observed despite differences in sowing times and cultivation environments. Hence, the validated method was successfully applied to various CSEs.

## 4. Conclusions

The HPLC method developed and optimized in this study is capable of concurrently analyzing flavonoids in CSE. Validation data, namely linearity, precision, percentage of recovery, LOD, and LOQ were found to be adequate for the intended purpose. The method was successfully used to quantify and qualify CSE on the basis of retention times and UV spectra. The developed method was very effective for the analysis of flavone-6-*C*-glycosides and flavone-8-*C*-glycosides in CSE. In addition, the method can also separate stereoisomers of flavonol glycosides bearing different sugars (glucose or galactose). The broad versatility and good efficiency is a valuable feature of this simultaneous analysis method. The utility of the method can be further extended to the simultaneous analysis of flavone and flavonol isomers present in food.

## Figures and Tables

**Table 1:** Effect of the mobile-phase composition on the separation of isomers in common buckwheat sprout extract.

Composition of Mobile Phase (Solvent A/Solvent B)	Resolution (*Rs*)				Symmetry Factor					
	Luteolin Derivatives ^1^	Apigenin Derivatives ^2^	Quercetin Derivatives ^3^		Orientin	Isoorientin	Vitexin	Isovitexin	Q3R ^4^	Rutin
Water/Methanol	1.23 ± 0.00 ^c,5^	3.66 ± 0.03 ^c^	0.00 ± 0.00 ^c^		n.d. ^6^	n.d.	n.d.	n.d.	n.d.	n.d.
Water/Acetonitrile	2.53 ± 0.03 ^a^	10.17 ± 0.06 ^b^	2.09 ± 0.04 ^a^		1.17 ± 0.04	1.18 ± 0.05	0.96 ± 0.01	0.99 ± 0.01	1.12 ± 0.05	1.05 ± 0.02
Acidic water ^7^/Methanol	1.19 ± 0.00 ^c^	3.71 ± 0.01 ^c^	0.00 ± 0.00 ^c^		n.d.	n.d.	n.d.	n.d.	n.d.	n.d.
Acidic water/Acetonitrile	1.87 ± 0.00 ^b^	10.30 ± 0.03 ^a^	1.93 ± 0.02 ^b^		1.03 ± 0.02	1.03 ± 0.01	0.99 ± 0.02	0.99 ± 0.01	1.02 ± 0.02	1.00 ± 0.02

^1^ Luteolin derivatives, orientin and isoorientin; ^2^ Apigenin derivatives, vitexin and isovitexin; ^3^ Quercetin derivatives, Q3R and rutin; ^4^ Q3R, quercetin-3-*O*-robinobioside; ^5^ Means with different superscripts in the same column indicate significant differences (*p* < 0.05) by Tukey’s test, ^6^ n.d., not detected; ^7^ Acidic water is water containing 0.1% (*v*/*v*) formic acid.

**Table 2:** Calibration curves constructed from standard flavonoid solutions.

Flavonoid	Range (μg mL^−1^)	Calibration Curve	Correlation Coefficient (R)
Orientin	6.25–200.00	y = 13074.0x + 3111.6	0.9999
Isoorientin	6.25–200.00	y = 13895.0x + 1876.0	0.9999
Vitexin	6.25–200.00	y = 10604.0x + 4006.4	0.9999
Isovitexin	6.25–200.00	y = 11450.0x + 6941.8	0.9999
Rutin	6.25–200.00	y = 7751.5x + 4289.6	0.9999

**Table 3:** Validation data for the simultaneous analysis of flavonoids in common buckwheat sprout extract using HPLC.

Flavonoid	t_R_^1^ (min)	RSD ^2^ (%) of t_R_	LOD ^3^ (μg mL^−1^)	LOQ ^4^ (μg mL^−1^)	Spiking Level (μg mL^−1^)	Accuracy (%)		Precision (RSD; %)	
						Recovery (*n* = 3)	Confidence Interval (95%)	Intra-Day Repeatability (*n* = 3)	Inter-Day Reproducibility (*n* = 3 × 3 Days)
Orientin	36.4	0.13	0.32	0.96	25	97.10	96.91–103.59	3.74	4.04
					50	102.96		3.78	3.13
					100	100.69		2.80	4.30
Isoorientin	37.5	0.14	0.09	0.26	25	97.05	96.87–103.54	3.33	3.56
					50	102.88		4.18	2.85
					100	100.69		2.67	4.35
Vitexin	42.4	0.12	0.26	0.77	25	96.67	96.48–103.83	3.84	4.55
					50	103.09		3.69	3.46
					100	100.70		2.72	4.22
Isovitexin	47.9	0.07	0.29	0.88	25	97.12	96.84–104.20	3.61	4.13
					50	103.60		3.76	3.44
					100	100.83		2.69	4.24
Rutin	52.1	0.10	0.42	1.28	25	97.25	96.97–103.90	5.21	5.40
					50	103.35		4.25	3.57
					100	100.71		2.96	4.03

^1^ t_R_, retention time. ^2^ RSD, relative standard deviation. ^3^ LOD, limit of detection. ^4^ LOQ, limit of quantification.

**Table 4:** Application of the developed HPLC method to common buckwheat sprout extracts.

		Orientin	Isoorientin	Vitexin	Isovitexin	Q3R ^1^	Rutin
t_R_^2^ (min)		36.4	37.5	42.4	47.9	51.1	52.1
Content (mg g^−1^ DW ^4^)	No. 1	6.63 ± 0.17 ^d,3^	12.63 ± 0.21 ^a^	5.90 ± 0.11 ^e^	10.48 ± 0.18 ^b^	3.50 ± 0.06 ^f^	9.98 ± 0.21 ^c^
	No. 2	6.99 ± 0.04 ^d^	13.37 ± 0.20 ^a^	6.69 ± 0.32 ^d^	11.62 ± 0.25 ^b^	4.12 ± 0.07 ^e^	10.53 ± 0.23 ^c^
	No. 3	7.02 ± 0.08 ^d^	13.48 ± 0.29 ^a^	6.89 ± 0.23 ^d^	12.14 ± 0.29 ^b^	3.69 ± 0.04 ^e^	10.74 ± 0.44 ^c^
	No. 4	7.64 ± 0.11 ^e^	14.11 ± 0.27 ^b^	9.50 ± 0.06 ^c^	16.59 ± 0.22 ^a^	3.42 ± 0.07 ^f^	8.60 ± 0.08 ^d^
	No. 5	8.16 ± 0.44 ^c^	15.24 ± 0.71 ^a^	7.93 ± 0.24 ^c^	13.83 ± 0.68 ^a^	4.56 ± 0.64 ^d^	12.18 ± 0.53 ^b^
	No. 6	8.90 ± 0.22 ^c^	16.67 ± 0.33 ^a^	9.84 ± 0.25 ^b^	17.09 ± 0.50 ^a^	3.65 ± 0.03 ^d^	9.22 ± 0.11 ^bc^
	No. 7	7.33 ± 0.20 ^c^	14.00 ± 0.62 ^a^	7.29 ± 0.26 ^c^	12.92 ± 0.44 ^a^	4.45 ± 0.25 ^d^	11.74 ± 0.44 ^b^
	No. 8	6.99 ± 0.77 ^b^	13.27 ± 1.28 ^a^	7.26 ± 0.72 ^b^	13.34 ± 1.02 ^a^	3.90 ± 0.46 ^c^	10.70 ± 1.05 ^b^
	No. 9	8.48 ± 0.26 ^d^	15.83 ± 0.46 ^b^	9.65 ± 0.32 ^c^	16.80 ± 0.50 ^a^	4.21 ± 0.13 ^e^	9.84 ± 0.28 ^c^

^1^ Q3R, quercetin-3-*O*-robinobioside. ^2^ t_R_, retention time. ^3^ Means with different superscripts in the same row indicate significant differences (*p* < 0.05) by Tukey’s test. ^4^ DW, dry weight.

## Supplementary Materials

The following are available online at https://www.mdpi.com/2304-8158/8/11/549/s1, Figure S1: Structures of flavonoids in common buckwheat sprouts, Table S1: Resolution of common buckwheat sprout extract analyzed at: (A) 20 °C, (B) 30 °C, and (C) 40 °C, Table S2. Resolution of common buckwheat sprout extract analyzed on flow rate of: (A) 0.6, (B) 0.8, and (C) 1.0 mL min^−1^.
