# Supplementary material for: Developing and Validating a Method for Separating Flavonoid Isomers in Common Buckwheat Sprouts Using HPLC-PDA
Source: Foods. 2019 Nov 4;8(11):549. doi: 10.3390/foods8110549 (PMC6915543; doi:10.3390/foods8110549)
Supplement: Supplementary file 1 [file foods-08-00549-s001.pdf]

Developing and validating a method for separating flavonoid isomers in common buckwheat sprouts using HPLC-PDA

Davin Jang et al.

Supplementary data

Figure S1. Structure of flavonoids in common buckwheat sprouts.

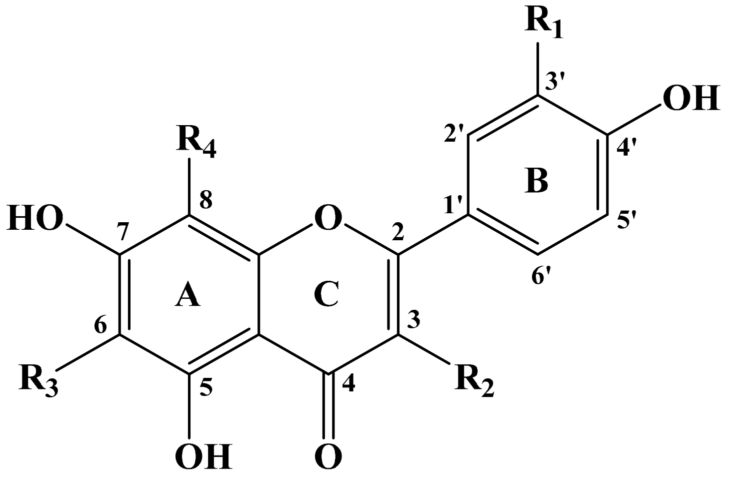

Compound	R_1	R_2	R_3	R_4
Orientin	OH	H	H	Glc [1]
Isoorientin	OH	H	Glc	H
Vitexin	H	H	H	Glc
Isovitexin	H	H	Glc	H
Quercetin-3-*O*-robinobioside (Q3R)	OH	-*O*-Gal [2]-Rha [3]	H	H
Rutin	OH	-*O*-Glc-Rha	H	H

[1] Glc, glucose; [2] Gal, galactose; [3] Rha, rhamnose.

Table S1. Resolution of common buckwheat sprouts extract analyzed at: (A) 20°C, (B) 30°C, and (C) 40°C.

Temperature (°C)	Resolution (*Rs*)		
	Luteolin Derivatives [1]	Apigenin Derivatives [2]	Quercetin Derivatives [3]
20	1.05 ± 0.10	10.83 ± 0.42	0.00 ± 0.00
30	1.58 ± 0.01	9.64 ± 0.15	1.15 ± 0.01
40	1.87 ± 0.00	10.30 ± 0.03	1.93 ± 0.02

[1]Luteolin derivatives, orientin and isoorientin; [2]Apigenin derivatives, vitexin and isovitexin; [3]Quercetin derivatives, Q3R and rutin.

Table S2. Resolution of common buckwheat sprouts extract analyzed on flow rate of: (A) 0.6, (B) 0.8, and (C) 1.0 mL min^{-1}.

Flow rate (mL min^{-1})	Resolution (*Rs*)		
	Luteolin Derivatives [1]	Apigenin Derivatives [2]	Quercetin Derivatives [3]
0.6	1.65 ± 0.01	10.80 ± 0.02	1.44 ± 0.00
0.8	1.88 ± 0.01	10.06 ± 0.04	1.89 ± 0.02
1.0	1.95 ± 0.01	10.89 ± 0.11	1.98 ± 0.00

[1]Luteolin derivatives, orientin and isoorientin; [2]Apigenin derivatives, vitexin and isovitexin; [3]Quercetin derivatives, Q3R and rutin.
